# Drug Interaction and Pharmacist

**DOI:** 10.4103/0975-1483.66807

**Published:** 2010

**Authors:** JA Ansari

**Affiliations:** *Department of Pharmacology, Faculty of Pharmacy, Hamdard University, New Delhi 110 062, India*

**Keywords:** Computerized screening systems, current indian scenario of drug interactions, drug interaction management, drug interactions

## Abstract

The topic of drug–drug interactions has received a great deal of recent attention from the regulatory, scientific, and health care communities worldwide. Nonsteroidal anti-inflammatory drugs, antibiotics and, in particular, rifampin are common precipitant drugs prescribed in primary care practice. Drugs with a narrow therapeutic range or low therapeutic index are more likely to be the objects for serious drug interactions. Object drugs in common use include warfarin, fluoroquinolones, antiepileptic drugs, oral contraceptives, cisapride, and 3-hydroxy-3-methylglutaryl coenzyme A reductase inhibitors. The pharmacist, along with the prescriber has a duty to ensure that patients are aware of the risk of side effects and a suitable course of action should they occur. With their detailed knowledge of medicine, pharmacists have the ability to relate unexpected symptoms experienced by patients to possible adverse effects of their drug therapy.

## INTRODUCTION

The topic of drug–drug interactions (DDIs) has received a great deal of recent attention from the regulatory, scientific, and health care communities worldwide.[1] A large number of drugs are introduced every year, and new interactions between medications are increasingly reported. Consequently, it is no longer practical for physicians to rely on memory alone to avoid potential drug interactions. Precipitant drugs modify the object drug’s absorption, distribution, metabolism, excretion, or actual clinical effect. Nonsteroidal anti-inflammatory drugs, antibiotics and, in particular, rifampin are common precipitant drugs prescribed in primary care practice. Drugs with a narrow therapeutic range or low therapeutic index are more likely to be the objects for serious drug interactions. Object drugs in common use include warfarin, fluoroquinolones, antiepileptic drugs, oral contraceptives, cisapride, and 3-hydroxy-3-methylglutaryl coenzyme A reductase inhibitors.[2] Serotonin syndrome is a potentially life-threatening disorder of excessive serotoninergic activity often due to drug interactions.[3] Many other drugs act as precipitants or objects, and a number of drugs act as both.[2] An overview of selected serious drug interactions is given in Table 1.[2]

**Table 1:** Overview of selected serious drug interactions[2]

Interaction	Potential effect	Time to effect	Recommendations and comments
Warfarin (Coumadin) *plus* ciprofloxacin (Cipro), clarithromycin (Biaxin), erythromycin, metronidazole (Flagyl) or trimethoprim- sulfamethoxazole (Bactrim, Septra)	Increased effect of warfarin	Generally within 1 week	Select alternative antibiotic
Warfarin *plus* acetaminophen	Increased bleeding, increased INR	Any time	Use lowest possible acetaminophen dosage and monitor INR
Warfarin *plus* acetylsalicylic acid (aspirin)	Increased bleeding, increased INR	Any time	Limit aspirin dosage to 100 mg per day and monitor INR
Warfarin *plus* NSAID	Increased bleeding, increased INR	Any time	Avoid concomitant use if possible; if coadministration is necessary, use a cyclooxygenase-2 inhibitor and monitor INR
Fluoroquinolone *plus* divalent/trivalent cations or sucralfate (Carafate)	Decreased absorption of fluoroquinolone	Any time	Space administration by 2–4 h
Carbamazepine (Tegretol) *plus* cimetidine (Tagamet), erythromycin, clarithromycin or fluconazole (Diflucan)	Increased carbamazepine levels	Generally within 1 week	Monitor carbamazepine levels
Phenytoin (Dilantin) *plus* cimetidine, erythromycin, clarithromycin or fluconazole	Increased phenytoin levels	Generally within 1 week	Monitor phenytoin levels
Phenobarbital *plus* cimetidine, erythromycin, clarithromycin or fluconazole	Increased phenobarbital levels	Generally within 1 week	Clinical significance has not been established.
			Monitor phenobarbital levels
Phenytoin *plus* rifampin (Rifadin)	Decreased phenytoin levels	Generally within 1 week	Clinical significance has not been established.
			Monitor phenytoin levels
Phenobarbital *plus* rifampin	Decreased phenobarbital levels	Generally within 1 week	Monitor phenobarbital levels
Carbamazepine *plus* rifampin	Decreased carbamazepine levels	Generally within 1 week	Clinical significance has not been established. Monitor carbamazepine levels
Lithium *plus* NSAID or diuretic	Increased lithium levels	Any time	Decrease lithium dosage by 50% and monitor lithium levels
Oral contraceptive pills *plus* rifampin	Decreased effectiveness of oral contraception	Any time	Avoid if possible. If combination therapy is necessary, have the patient take an oral contraceptive pill with a higher estrogen content (>35 µg of ethinyl estradiol) or recommend alternative method of contraception
Oral contraceptive pills *plus* antibiotics	Decreased effectiveness of oral contraception	Any time	Avoid if possible. If combination therapy is necessary, recommend use of alternative contraceptive method during cycle
Oral contraceptive pills *plus* troglitazone (Rezulin)	Decreased effectiveness of oral contraception	Any time	Have the patient take an oral contraceptive pill with a higher estrogen content or recommend alternative method of contraception
Cisapride (Propulsid) *plus* erythromycin, clarithromycin, fluconazole, itraconazole (Sporanox), ketoconazole (Nizoral), nefazodone (Serzone), indinavir (Crixivan) or ritonavir (Norvir)	Prolongation of QT interval along with arrhythmias secondary to inhibited cisapride metabolism	Generally within 1 week	Avoid. Consider whether metoclopromide (Reglan) therapy is appropriate for the patient
Cisapride *plus* class IA or class III antiarrhythmic agents, tricyclic antidepressants or phenothiazine	Prolongation of QT interval along with arrhythmias	Any time	Avoid. Consider whether metoclopromide therapy is appropriate for the patient
Sildenafil (Viagra) *plus* nitrates	Dramatic hypotension	Soon after taking sildenafil	Absolute contraindication
Sildenafil *plus* cimetidine, erythromycin, itraconazole or ketoconazole	Increased sildenafil levels	Any time	Initiate sildenafil at a 25-mg dose
HMG-CoA reductase inhibitor *plus* niacin, gemfibrozil (Lopid), erythromycin or itraconazole	Possible rhabdomyolysis	Any time	Avoid if possible. If combination therapy is necessary, monitor the patient for toxicity
Lovastatin (Mevacor) *plus* warfarin	Increased effect of warfarin	Any time	Monitor INR
SSRI *plus* tricyclic antidepressant	Increased tricyclic antidepressant level	Any time	Monitor for anticholinergic excess and consider lower dosage of tricyclic antidepressant
SSRI *plus* selegiline (Eldepryl) or nonselective monoamine oxidase inhibitor	Hypertensive crisis	Soon after initiation	Avoid
SSRI *plus* tramadol (Ultram)	Increased potential for seizures; serotonin syndrome	Any time	Monitor the patient for signs and symptoms of serotonin syndrome
SSRI *plus* St. John’s wort	Serotonin sytidrome	Any time	Avoid
SSRI plus naratnptan (Amerge), rizatriptan (Mazalt), sumatriptan (Imitrex) or zolmitriptan (Zomig)	Serotonin sytidrome	Possibly after initial dose	Avoid if possible. If combination therapy is necessary, monitor the patient for signs and symptoms of serotonin syndrome

INR, International Normalized Ratio; NSAID, nonsteroidal anti-inflammatory drug; HMG-CoA, 3-hydroxy-3-methylglutaryl coenzyme A reductase inhibitor; SSRI, selective serotonin reuptake inhibitor

## SERIOUSNESS AND SEVERITY OF DRUG INTERACTION

The American Food and Drug Administration define a serious adverse event as one when the patient outcome is one of the following[4]:

- Death
- Life-threatening
- Hospitalization (initial or prolonged)
- Disability—significant, persistent, or permanent change, impairment, damage or disruption in the patient’s body function/structure, physical activities, or quality of life.
- Congenital anomaly
- Requires intervention to prevent permanent impairment or damage

Severity is a point on an arbitrary scale of intensity of the adverse event in question. The terms “severe” and “serious” when applied to adverse events are technically very different. They are easily confused but cannot be used interchangeably, require care in usage. A headache is severe, if it causes intense pain. There are scales such as “Visual Analog Scale” that helps us assess the severity. On the other hand, a headache can hardly ever be serious, unless it also satisfies the criteria for seriousness listed above.

## MECHANISMS

As research better explains the biochemistry of drug use, fewer ADRs (adverse drug reactions) are Type B and more are Type A. Common mechanisms are:

Abnormal pharmacokinetics due to

- genetic factors
- comorbid disease states

Synergistic effects between either

- a drug and a disease
- two drugs

### Abnormal pharmacokinetics

#### Comorbid disease states

Various diseases, especially those that cause renal or hepatic insufficiency, may alter drug metabolism. Resources are available that report changes in a drug’s metabolism due to disease states.[5]

#### Genetic factors

Abnormal drug metabolism may be due to inherited factors of either Phase I oxidation or Phase II conjugation.[6,7] Pharmacogenomics is the study of the inherited basis for abnormal drug reactions.

### Phase I reactions

Inheriting abnormal alleles of cytochrome P450 can alter drug metabolism. Tables are available to check for drug interactions due to P450 interactions.[8,9]

Inheriting abnormal butyrylcholinesterase (pseudocholinesterase) may affect metabolism of drugs such as succinylcholine.[10]

### Phase II reactions

Inheriting abnormal N-acetyltransferase which conjugated some drugs to facilitate excretion may affect the metabolism of drugs such as isoniazid, hydralazine, and procainamide.[9,10] Inheriting abnormal thiopurine S-methyltransferase may affect the metabolism of the thiopurine drugs mercaptopurine and azathioprine.[9]

#### Interactions with other drugs

The risk of drug interactions is increased with polypharmacy.

### Protein binding

These interactions are usually transient and mild until a new steady state is achieved. These are mainly for drugs without much first-pass liver metabolism. The principal plasma proteins for drug binding are[11]:

- albumin
- α1-acid glycoprotein
- lipoproteins

Some drug interactions with warfarin are due to changes in protein binding.[11]

### Cytochrome P450

Patients have abnormal metabolism by cytochrome P450 due to either inheriting abnormal alleles or drug interactions. Tables are available to check for drug interactions due to P450 interactions.[8]

### Synergistic effect

An example of synergism is two drugs that both prolong the QT interval.

## MANAGEMENT OF DRUG INTERACTION

### The role of pharmacogenetics and pharmacogenomics[12]

An individual’s genetic makeup can alter their response to a drug. Genetics affect pharmacokinetics and pharmacodynamics. Unrecognized mutations can be associated with ADRs or can affect the magnitude of a drug interaction. A common example is the metabolism of ethanol. There are ethnic differences in the metabolism of ethanol by alcohol dehydrogenase. People of Chinese descent have a higher incidence of atypical alcohol dehydrogenase and therefore become flushed and dizzy when they consume alcohol. Their capacity for consuming alcohol is lower than that for other populations.

To apply pharmacogenetics and pharmacogenomics to the management of drug interactions, it is important to know the difference between the two terms. Pharmacogenetics applies to inherited traits and genetic polymorphisms. Polymorphism refers to stable allelic variations found in the population (occurring at a frequency >1%) that result in altered protein activity. Pharmacogenomics applies to the entire spectrum of genes. With pharmacogenetics, the focus is on metabolizing enzymes and transporters, whereas with pharmacogenomics, the focus is on individualized drug and dosage for a specific disease.

### The role of pharmacist in management of drug interaction

The pharmacist, along with the prescriber has a duty to ensure that patients are aware of the risk of side effects and a suitable course of action should they occur. With their detailed knowledge of medicine, pharmacists have the ability to relate unexpected symptoms experienced by patients to possible adverse effects of their drug therapy. The practice in clinical pharmacy also ensures that ADRs are minimized by avoiding drugs with potential side effects in susceptible patients. Thus, pharmacist has a major role to play in relation to prevention, detection, and reporting ADRs.[13]

### Management options of drug interaction include

*Avoiding the combination entirely:* For some drug interactions, the risk always outweighs the risk, and the combination should be avoided. Because drug classes are usually heterogeneous with regard to drug interactions (as described above), one can often select a no interacting alternative for either the object drug or the precipitant drug.[14]

*Adjusting the dose of the object drug:* Sometimes, it is possible to give the two interacting drugs safely as long as the dose of the object drug is adjusted.

*Spacing dosing times to avoid the interaction:* For some drug interactions involving binding in the gastrointestinal tract, to avoid the interaction one can give the object drug at least 2 h before or 4 h after the precipitant drug. In this way, the object drug can be absorbed into the circulation before the precipitant drug appears.

*Monitoring for early detection:* In some cases, when it is necessary to administer interacting drug combinations, the interaction can be managed through close laboratory or clinical monitoring for the evidence of the interaction. In this way, the appropriate dosage changes can be made, or the drugs discontinued if necessary.

*Provide information on patient risk factors that increases the chance of an adverse outcome:* It is clear from the clinical experience of physicians and pharmacists as well as published studies that most patients who take interacting drug combinations do not manifest adverse consequences.[15] Substantial evidence from both the clinical experience of physicians and pharmacists as well as published studies suggest that the risk of statin-induced myopathy increases with increasing serum concentrations of the statin. Accordingly, it has been recommended that simvastatin should not exceed 20 mg daily in patients receiving verapamil concurrently.[16]

*Improve computerized screening systems:* It is clear that computerized drug interaction screening systems have not been as successful as one hoped.[14,17]

*Excessive number of drug interactions on the systems:* Many pharmacists find that computerized drug interaction screening systems detect a large number of DDIs of questionable clinical significance.

*Drug class differences not handled correctly:* Almost all drug classes interact heterogeneously, because individual members of a drug class are often not metabolized by the same cytochrome P450 isozymes or ABC (ATP-binding cassette) transporters as other members of the class. The statins are a good example, because simvastatin and lovastatin are extensively metabolized by CYP3A4, atorvastatin is moderately metabolized by CYP3A4, fluvastatin is metabolized by CYP2C9, and pravastatin and rosuvastatin are not metabolized by cytochrome P450 isozymes.[18] Thus, combining all members of this drug class together is rarely justified when considering drug interactions. Nonetheless, it is common for reviews and computer systems to include all statins together as interacting with CYP3A4 inhibitors, even though the risk is primarily limited to lovastatin, simvastatin, and to a lesser extent, atorvastatin.[19]

## CURRENT INDIAN SCENARIO OF DRUG INTERACTIONS AND ITS MANAGEMENT

The prescribing information for most drugs contains a list of potential drug interactions. Many of the listed interactions may be rare, minor, or only occur under specific conditions and may not be important. Drug interactions that cause important changes in the action of a drug are of the greatest concern.

Drug interactions are complex and chiefly unpredictable. A known interaction may not occur in every individual. This can be explained because there are several factors that affect the likelihood that a known interaction will occur. These factors include differences among individuals in their;[20,21]

- genes,
- physiology,
- age,
- lifestyle (diet, exercise),
- underlying diseases,
- drug doses,
- the duration of combined therapy, and
- the relative time of administration of the two substances (Sometimes, interactions can be avoided if two drugs are taken at different times).

Nevertheless, important drug interactions occur frequently and they add millions of dollars to the cost of health care. Moreover, many drugs have been withdrawn from the market because of their potential to interact with other drugs and cause serious health care problems.

### Management[22]

- Before starting any new prescription drug or over-the-counter drug, talk to your primary health care provider or pharmacist. Make sure that they are aware of any vitamins or supplements that you take.
- Make sure to read the patient information handout given to you at the pharmacy. If you are not given an information sheet, ask your pharmacist for one.
- Check the labels of your medications for any warnings and look for the “Drug Interaction Precaution.” Read these warnings carefully.
- Make a list of all your prescription medications and over-the-counter products, including drugs, vitamins, and supplements. Review this list with all health care providers and your pharmacist.
- If possible, use one pharmacy for all your prescription medications and over-the-counter products. This way your pharmacist has a record of all your prescription drugs and can advise you about drug interactions and side effects.

This brief overview of drug interactions does not cover every possible scenario. Individuals should not be afraid to use their drugs because of the potential for drug interactions. Rather, they should use the information that is available to them to minimize the risk of such interactions and to improve the success of their therapy.

## SOME GUIDELINES FOR COMMUNITY PHARMACIST[20,21]

Performing or obtaining necessary assessments of the patient’s health status is as follows:

- Formulating a medication treatment plan: selecting, initiating, modifying, or administering medication therapy.
- Monitoring and evaluating the patient’s response to therapy, including safety and effectiveness.
- Performing a comprehensive medication review to identify, resolve, and prevent medication-related problems, including adverse drug events.
- Documenting the care delivered and communicating essential information to the patient’s other primary care providers.
- Providing verbal education and training designed to enhance patient understanding and appropriate use of his or her medications.
- Providing information, support services, and resources designed to enhance patient adherence with his or her therapy.
- Coordinating and integrating medication therapy management services within the broader health care-management services being provided to the patient.
- Give health care practitioners a complete list of all of the drugs that you are using or have used within the last few weeks. This should include over-the-counter medications, vitamins, food supplements, and herbal remedies.
- Inform health care practitioners when medications are added or discontinued.
- Inform health care practitioners about changes in lifestyle (for example, exercise, diet, alcohol intake).
- Ask your health care practitioners about the most serious or frequent drug interactions with the medications that you are taking.
- Since the frequency of drug interactions increases with the number of medications, work with your health care practitioners to eliminate unnecessary medications.

## CONCLUSION

The past several years have seen major advances in our understanding of DDIs, particularly in the area of the molecular mechanisms by which drug interact. However, our ability to appropriately apply this information to specific patients has lagged far behind. Pharmacists must take responsibility for monitoring for drug interactions and notifying the physician and patient about potential problems.
